# Identifying port maritime communities: application to the Spanish case

**DOI:** 10.1186/s12544-021-00495-1

**Published:** 2021-06-22

**Authors:** Nicanor García, Belarmino Adenso-Díaz, Laura Calzada-Infante

**Affiliations:** 1 Port Authority of Gijon, Gijón, Spain; 2 Escuela Politécnica de Ingenieros, Campus de Viesques, E-33204 Gijón, Spain

**Keywords:** Maritime traffic, Port communities, Spanish port system, Bipartite networks, Port competition, Port collaboration

## Abstract

The aim of this paper is to detect port maritime communities sharing similar international trade patterns, by a modelisation of maritime traffic using a bipartite weighted network, providing decision-makers the tools to search for alliances or identify their competitors. Our bipartite weighted network considers two different types of nodes: one represents the ports, while the other represents the countries where there are major import/export activity from each port. The freight traffic among both types of nodes is modeled by weighting the volume of product transported. To illustrate the model, the Spanish case is considered, with the data segmented by each type of traffic for a fine tuning. A sort of link prediction is possible, finding for those communities with two or more ports, countries that are part of the same community but with which some ports do not have yet significant traffic. The evolution of the traffics is analyzed by comparing the communities in 2009 and 2019. The set of communities formed by the ports of the Spanish port system can be used to identify global similarities between them, comparing the membership of the different ports in communities for both periods and each type of traffic in particular.

## Introduction

Maritime transport, which is responsible for around four-fifths of the world merchandise trade traffic, has proved to be the backbone of globalised trade and the manufacturing supply chain. Before the global economic and health crisis of COVID-19, in the last global report, forecasts for the period 2019–2024 predicted an increase of 3.4% for maritime transport in that period, with 11 billion tonnes and an estimated maritime trade of 793.26 million TEUs handled in container ports worldwide with the following distribution: 64% in Asia, 16% in Europe, 8% in North America, 7% in Latin America and the Caribbean, 4% Africa, and 2% Oceania [7]. In such a competitive and changing environment, it is critical to know the strengths available and all the information that will allow the actors involved in maritime transport to make the right decisions.

The efficiency improvement in the port sector has been widely studied using various approaches such as data envelopment analysis (DEA) or stochastic frontier analysis (SFA), concluding that the information obtained assist maritime stakeholders’ decision-making [18]. However, comparing ports within a given geographical scope has been less studied, as usually connections among ports are the objects of the study, and connections are not restricted to geographical closeness [16, 46].

In this regard, identification of ports with similar characteristics, such as commercial partners, location, technological skills or the learning and experience curve advantages over rivals, could help to identify possible ways of collaboration among them as well as to understand differentiating factors concerning other competing ports [60]. However, this identification process not only requires a great knowledge of the port environment, but also the use of high-level resources, involving both technological tools and functional consultancy which are not always at the disposal of all ports. These tools may also have shortcomings, as they might not take into account all the aspects and variables involved in the categorisation of port infrastructures. This is why it is necessary to find tools to help port managers in the process of identifying port infrastructures with which they can be compared.

In addition, this identification process is not trivial given the lack of knowledge about other facilities, and the fact that research in maritime transport has been far less studied than other modes of transport, especially from a network perspective [20]. Many of the existing studies of this type are theoretical, based on simulation due to this lack of data [21]. One of the most common sources of information on which studies on maritime traffic are usually based are the annual reports and traffic published by each port infrastructure, as well as commercial tools that are not always available for the researchers.

Both in the scientific literature and in consulting studies, comparisons between different ports are usually established based on the exploitation variables of the said ports, or in terms of traffic comparisons, without taking into account a detailed analysis of the traffic of similar ports. As mentioned, the lack of data for decision-making and the difficulty of identifying ports with similar characteristics for a given type of traffic (either import or export flows) makes this approach hard.

This article proposes a novel method for grouping ports and countries with similar traffic patterns, which may serve as a useful tool for port managers to establish the most suitable collaboration or competition strategies with other port infrastructures. The proposed methodology for identifying the grouping of ports is based on Complex Network Analysis (CNA), a modeling tool that in recent years has been increasing its use in maritime studies [2]. The use of CNA techniques would allow to find groups of ports and countries, and within these “communities” (a.k.a. clusters), ports in the same group are those that have very similar markets and ultimately are fighting for similar cargoes. With this information it is possible to identify countries in the same community rather than a specific port but not having yet significant traffic with it. This “link prediction” process allows port managers to identify competing infrastructures, potential partners and countries where they are potentially competing for some specific commodities.

This paper is structured as follows: Section two reviews the most relevant literature, both on port strategy and on complex networks analysis. Section three explains the methodology followed and describes the dataset used. Section four describes some results for export and import flows along with the communities formed by these flows, and sections five and six are devoted to discussion and summarising the final conclusions.

## Relevant literature

Given the global struggle for markets in maritime transport, it is not surprising that competition between ports has attracted the attention of scientific literature for over four decades with increasing interest since the late 1990s [41].

Competition between ports has been studied from multiple points of view such as the impact of competition on performance or the institutional competitive pressure [15], their hinterland accessibility [30] or using a Social Network Analysis (SNA) approach to discuss the research trends [37].

Ports are part of a supply chain in which the stakeholders and their preferences are very heterogeneous. In this context, it is impossible to understand the decision-making processes of each actor without considering that these processes are all related to each other [14].

Depending on the geographical location and the services offered, the degree of competence may differ [8] and in certain circumstances, a certain degree of collaboration may be more interesting than competition among different ports. This is the case of the adjacent ports, where it is particularly interesting to study the possibilities of competition and cooperation between them [47]. There are numerous examples in the scientific literature of cooperation between ports, such as the ports of Seattle and Tacoma, located 30 miles from one another [68], North Adriatic ports [56] or even cooperation in port investment between liner companies and ports [55].

There are also interesting examples that identify Port Cooperation Policies and homogeneous groups of ports in the Mediterranean region through traditional clustering techniques [28], port cooperation in connection with the governance structure of ports in Japan [53], port collaboration strategies in China [34, 57] and papers considering possible combination of both cooperative and non-cooperative strategies among ports [36]. Different market scenarios will lead to varying types of strategy, competition or cooperation. Li et al. [42] consider that when resources are abundant, more cooperation between ports will emerge, as this environment will encourage these phenomena given the advantages that cooperation will bring to ports in the face of competition.

Due to the rapid changes in the maritime sector, several strategies have emerged to quickly adapt to these changes, such as coopetition, whereby ports compete and cooperate simultaneously to achieve objectives of common interest among the actors involved [54]. Coopetition may vary according to different combinations of terminal ownership [39] and may be especially suitable in uncertain scenarios [64].

Although they are not too frequent, examples can be found of studies that look for similarities between different players in the port business, or propose classifications that allow the ranking of port facilities such as container terminals in a certain area [1]. There are also examples of comparisons of port facilities in a certain geographical area, such as the study of the impact of large infrastructure projects in the port choice in the case of Colombia [62]. Other research studies have taken as reference data from the same geographical unit of this paper (NUTS-3), examining the spatial distribution of flows between the French NUTS-3 regions and the ports of Western Europe [31].

The reasons and policies to promote cooperation and integration of ports have been widely studied including the challenges in Port Integration, practices and models for Port Cooperation and the impediments for these kinds of efforts [48]. There are examples of studies in local environments on the preferences between cooperation and collaboration of neighboring ports, recommending proactive strategies of cooperation in the case study of Chilean ports [61]. The appropriate strategy for each case will depend on the special conditions of each environment and that is why the decision-makers will need to have as much information as possible in order to pursue collaborative or competitive strategies.

Most papers using that approach define the ports as nodes with links representing sea lines [24, 43], or other concepts related to maritime connections [19, 22, 45, 63].

A special type of network, the bipartite network, considers two different types of nodes in the graph. Examples of bipartite networks are found in modeling the people and social groups they belong to, the musical artists and musical genres they play, and the text documents and words they contain among others [32, 40].

Some other papers also define nodes representing ports and countries with which these ports have flows of import and export freight, being these flows the edges of the network. These studies have focussed on researching the connectivity to international markets of the countries [12], the bilateral connectivity in the liner shipping network [6], the vulnerability of international freights [13], and container shipping [66].

Although not particularly common, examples can be found in the literature on the study of communities in maritime traffic (i.e., ports strongly connected among them), such as the use of these communities to find co-operative networks and ‘hidden families’ in the container port industry [49], the search of community structures in cargo flows [38], maritime shipping networks [29, 58], and the seminal paper to find communities in maritime networks [23], which quoted the identification of tightly connected ports and the identification of bridge ports as a useful tool to “address inter-port cooperation”.

## Methodology

### Communities identification

A large number of clustering algorithms were defined, allowing the identification of communities in complex networks, that is, groups of nodes more closely connected among them (according to a particular measurable rule) than connected to the rest of the network. In the case of bipartite networks there are different methods for finding the communities structure of the graphs, some of them grouping only one type of node and other approaches forming communities in which both types of nodes are grouped together [65]. Extant algorithms are grouped into Modularity-Based Algorithms, Label Propagation Algorithms and Statistical Modelling and Minimum Description Length based (MDL) Algorithms [3].

Although it is possible to apply these kinds of algorithms directly to our dataset, experiments on real-world bipartite networks show that random walk based algorithms such as Louvain (a modularity-based algorithm) and Infomap (MDL algorithm) are more functional in detecting the communities in bipartite networks than the aforementioned algorithms [4]. The latter usually offers better results in bipartite networks applying it over its projected network [3, 67]. The projected network represents only one type of node, keeping the information of the whole network with its links, as Infomap works properly with weighted directed networks. For these reasons, Infomap has been the chosen algorithm to create communities for our data model.

The original concept of Infomap was developed by Rosvall & Bergstrom [52]. Multiple implementations of Infomap algorithm can be found in Bohlin et al. [11]. Infomap algorithm encodes the random walks, giving names to the nodes using the Huffman code [33]. The length of each code depends on the frequency that the random walk goes through, it being shorter for the nodes which are visited more frequently.

For each partition, it measures the description length in the map equation that comprises the entropy of the movements between the modules, and in the modules. The space of possible solutions is explored by a greedy search algorithm and refined with a simulated annealing approach that minimises the description length in the map equation.

### Dataset

As a testbed for our approach to detect port communities, we have considered data corresponding to the Spanish foreign trade hauls through maritime ports. The Spanish State-owned Port System is made up of 46 ports of national interest, managed by 28 Port Authorities, whose coordination corresponds to the Public Agency “Puertos del Estado”, which is responsible for executing the Government’s port policy. The activity of the state port system contributes nearly 20% of the GDP of the transport sector, representing 1.1% of the Spanish GDP and generating more than 35,000 direct jobs and about 110,000 indirect jobs [51].

Given the geographic particularity of the country, for some Spanish regions Portuguese ports are better connected and are more convenient for movement of their goods than using the national infrastucture. In that sense, two Portuguese groups of ports (namely Lisbon and Leixões) are being partialy considered as part of the ports system studied here, only in what refers to Spanish origin/destination cargo. When there is evidence that Spanish import/export traffic is routed through these Portuguese ports, they are being included in the data set.

The above mentioned ports, as European ports, are integrated in the Trans-European Transport Network (TEN-T), which includes, in addition to the port infrastructures, railway lines, roads, inland waterways, maritime shipping routes, airports and railroad terminals. TEN-T Network comprises two network layers, namely “The Core Network” with the most important connections, initially composed of 83 ports, to be completed by 2030 and the Comprehensive Network covering all European regions with an initial number of 236 ports, to be completed by 2050 [26].

For this study, data are gathered from the database “Bases Portuarias” [9] that covers the 2009–2019 time period. Goods are grouped according to the criteria established by the European Union to regulate the codes of customs declarations [25], which is used by all European ports to categorise their traffic.

**Fig. 1:**
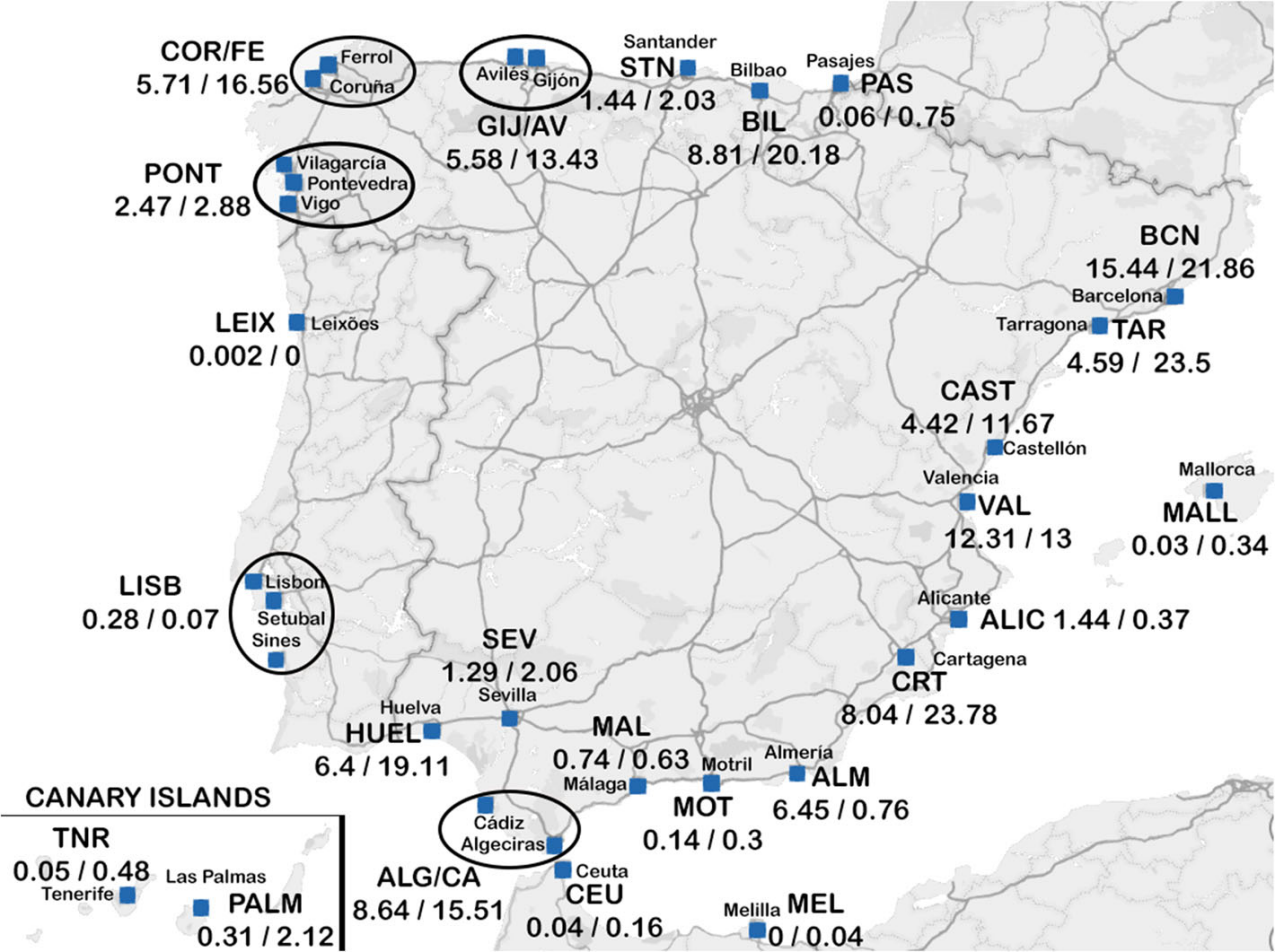
Location of the ports studied, and global Exports / Imports in trillions of Tonnes. Ports grouped due to their closeness are represented within an ellipse

The ports in the dataset are grouped by their province of origin NUTS-3 as described in [27]. Ports located in the same province are grouped in one unique node. This is for instance the case of the Port Authority of Vigo, Port Authority of Marín-Pontevedra and Port Authority of Vilagarcía, all located in Pontevedra province that will be referred to as “Pontevedra” in the text. The same applies for ports “Coruña/Ferrol” in Coruña province, “Gijón/Avilés” in Asturias, and the ports of “Algeciras/Cádiz”. The Portuguese ports of Lisbon, Setubal and Sines are kept grouped as the port of “Lisboa”. In some cases abbreviations and codes will be used to refer to the above-mentioned ports (see Fig. 1).

Data are grouped by the nature of the transported product (38 categories), according to nine statistical groups (Table 1) and the flow direction (import/export). For each product and flow direction, a bipartite network is defined and when there is a trade between a port and a country, this relationahip is modeled by a link in the corresponding network. Due to the large number of types of links with a residual traffic (with few tonnes transported), those links with a contribution of less than 1% of the total traffic for a port are not considered as well as all those port-country links whose contribution per type of traffic is less than 1% in the national traffic. With this criterium each port conserves the relevant links for each type of traffic. The number of links for import and export for each category of traffic in 2019 are detailed in Table 1. The total number links for the available years and for the import and export flows is detailed in Table 2. It can be noted on one hand, a clear upward trend in the number of links for export flows, which have increased by more than 50% from 2009 to 2019, and on the other hand, a slight growth in links for import traffic.

**Table 1:** Number of Export/Import links in the bipartite graphs corresponding to each type of product, in 2019

Statistical Group	Category	Export / Import links	Group Total Export / Import	Total Export / Import
1.- Energy	1.- Crude Oil	14/115	1915 / 1018	28,843 / 15,964
	12.- Coal and petroleum coke	129/128		
	2.- Fueloil	77/74		
	3.- Diesel fuel	100/68		
	35.- Natural gas	12/47		
	4.- Gasoline	229/78		
	51.- Biofuels	675/230		
	6.- Other petroleum products	625/224		
	7.- Energy gases from petroleum	54/54		
2.- Steelworks	10.- Other minerals and metal waste	362/347	2844 / 1856	
	11.- Iron scrap	72/153		
	13.- Steel products	1458/816		
	36.- Other metallurgical products	934/520		
	8.- Iron Ore	18/20		
3.- Non-Metallic Ores	25.- Regular salt	270/53	955 / 328	
	52.- Other Non-Metallic Ores	685/275		
4.- Fertilizers	14.- Phosphates	4/14	702 / 409	
	15.- Potash	106/56		
	16.- Natural and artificial fertilizers	592/339		
5.- Chemical Products	17.- Chemical products	2758/1285	2758 / 1285	
6.- Construction Materials	18.- Cement and clinker	205/44	2699 / 1015	
	20.- Elaborated construction materials	2365/932		
	5.- Asphalt	129/39		
7.- Agricultural and Food	21.- Cereals and their flours	397/319	7689 / 4044	
	22.- Soya beans	9/42		
	23.- Fruits, vegetables, legumes	746/548		
	24.- Wines, beverages, alcohols and derivatives	1403/380		
	27.- Canned food	1042/428		
	28.- Tobacco, cocoa, coffee, spices	742/504		
	29.- Oils and Fats	969/303		
	30.- Other food products	1287/580		
	33.- Frozen and refrigerated fish	368/573		
	37.- Feed and forage	726/367		
8.- Other Goods	19.- Wood and cork	705/400	7653 / 5109	
	26.- Paper and pulp	871/427		
	31.- Machine tool spare parts	2832/1773		
	34.- Rest of goods	3245/2509		
9.- Vehicles and Transport Elements	32.- Automobiles and their Parts	1628/900	1628 / 900

**Table 2:** Number of links Port-Country in the bipartite networks, for import and export flows in period 2009–2019

**Year**	2009	2010	2011	2012	2013	2014	2015	2016	2017	2018	2019
**Export**	18,534	22,095	24,580	23,282	26,285	27,188	27,990	28,936	29,581	28,572	28,238
**Import**	13,654	13,736	13,470	13,766	14,079	14,768	15,040	15,394	15,562	15,645	15,583

The sum of the tonnes transported between the port and the country in the case of export (or vice versa in the case of the import flow), is the weight of the edges in the bipartite networks, in millions of tonnes. With these input data (port, country and the tonnes of product moved), it is possible to create a bipartite weighted network for the future identification of the communities formed by the nodes that comprise it.

The next step is to export these data into R [59] where the Infomap algorithm is implemented [17]. The input data correspond to the tuples port-country-millions of tonnes moved, are used by R to call *infomap* for the calculation of the communities. Once the calculations are made, all the results are exported to Gephi [10] for a more accurate visualisation.

## Results

To illustrate the process, given the large amount of traffic categories, some products having a major impact on the GDP in 2019 [35] have been selected for a detailed analysis. According to Table 3, Automobiles and their components, as well as fuels (Biofuels in this case) became good candidates for this selection. Although they do not correspond exactly to the classification of the products of the dataset of origin, they do coincide for the most part, especially in the goods ranked on the top positions.

**Table 3:** Products with the greatest economic impact on Spanish gross domestic product in 2019. Source: [35]

Export		Import	
Product	M€	Product	M€
1. Automobiles	37,351	1. Chemical Products	44,866
2. Chemical Products	33,627	2. Fuels and Lubricants	42,657
3. Automobile Components	18,293	3. Automobile Components	24,846
4. Fuels and Lubricants	16,584	4. Automobiles	22,100
5. Textile Garments	11,806	5. Textile Garments	16,007
6. Foundry and steel products	11,195	6. Electronics and computers	14,697
7. Other unprocessed products	82,017	7. Foundry and steel products	11,022
8. Fresh and frozen fruits	7882	8. Electrical equipment	6981
9. Fresh Vegetables	6387	9. Fish and Seafood	5682

### Export

#### Automobiles and their parts

Automobile export traffic occupies the first place in the ranking of traffic positions with the greatest impact on the Spanish gross domestic product (37,351 M€). According to official data from the sector’s employers [5] in 2019 the automobile sector contributed 8.5% of the Spanish gross domestic product and 9% of the total employment of the active population that year, with the production of 2.8 millions of vehicles, of which 2.3 (81.84%) have been exported, accounting for 12.4% of the total exports from Spain.

The geographical proximity of some of the ports (as in the case of Vigo where PSA group is located, or Valencia with the Ford group) to the assembly factories of certain car brands, means that most cars manufactured in these facilities are exported through the geographically closest port, thus being a captive traffic. However, in the case of automobile factories geographically distant from the ports through which they can export, there may be competition among them for this type of traffic and the port that provides competitive advantages over the others will be able to move a greater quantity of products.

**Fig. 2:**
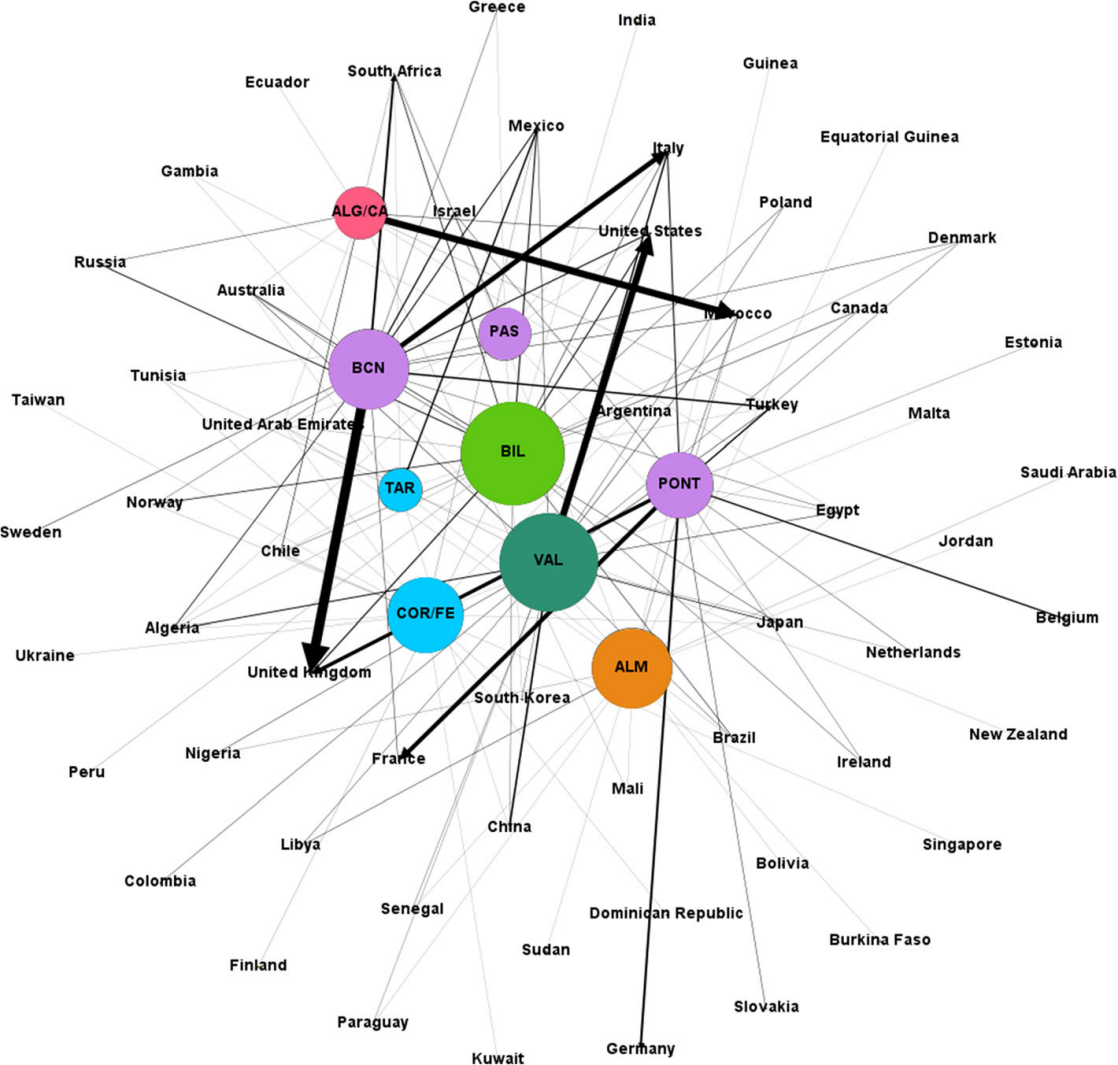
Main export links for automobiles and their parts in 2019. Nodes and arrows are proportional to the degree of the node and the weight of the link respectively. Nodes in the same community are represented with the same color

According to our dataset, the ports of Barcelona, Valencia, Pontevedra province, Bilbao and Algeciras/Cádiz occupy the first five positions for automobile traffic. These five sets of ports account for more than 90% of the total of this type of traffic. The most important port-contry links are between Barcelona-UK, Algeciras/Cádiz-Morocco, Valencia-United States, Barcelona-Italy and Pontevedra-France (Fig. 2).

Looking for the communities in the bipartite graph, six clusters are formed (Table 4) with the ports of Barcelona, Pontevedra and Pasajes sharing cluster #1 (along with 14 countries that constitute their main export destinations for the Automobile traffic) and the ports of Tarragona and Coruña/Ferrol sharing cluster #5. The other clusters are composed of a single port.

**Table 4:** Communities and link prediction for the “Automobiles and their parts” export graph, in 2019. Size means number of ports + number of countries in the community

Cluster #	Size	Ports	Missed potential markets
**1**	**3 + 14**	**Barcelona**	Belgium, Estonia, Germany, Netherlands, Slovakia.
		**Pontevedra**	Greece, South Africa, Sweden.
		**Pasajes**	Belgium, Denmark, Estonia, France, Germany, Netherlands, Slovakia, Sweden, Turkey, United Kingdom.
**2**	**1 + 9**	**Valencia**	
**3**	**1 + 12**	**Bilbao**	
**4**	**1 + 4**	**Algeciras/Cádiz**	
**5**	**2 + 9**	**Tarragona**	Dominican Republic, Kuwait, Malta, Singapore, Taiwan, Ukraine.
		**Coruña/Ferrol**	New Zealand
**6**	**1 + 10**	**Almería**	

In those communities with more than one port (such as #1 and #5), not all the countries in the community show significant trade of that product with all the ports in the cluster. This means for a specific port that other ports in the cluster are exporting to countries it is not trading with. This is a sort of “link prediction” that could be useful to find potential markets that are expected for those ports.

#### Biofuels

The export of this type of traffic is clearly dominated by the port of Barcelona, taking up more than half of the total traffic of the Spanish port system for this type of commodity, followed at a great distance by the ports of Valencia, Algeciras/Cádiz, Bilbao and Huelva.

In 2019 the port of Barcelona presented a very significant growth of this type of traffic, 92.2%, which made it reach 1.4 million tonnes [50] taking most of the overall increase in biofuels within the Spanish port system from 5 million tonnes in 2018 to 6.5 million tonnes in 2019.

Despite the clear dominance of Barcelona in this traffic, the relative uniformity of export traffic to certain countries, makes Infomap detect a large community formed by five ports, namely Barcelona, Cartagena, Algeciras/Cádiz, Castellón and Coruña/Ferrol, altogether with nine countries (Table 5). Regarding the rest of the detected communities is remarkably the case of the port of Valencia, which is not grouped with any other port but the algorithm includes it in a large community with 17 countries, which gives an idea of the dispersion of its traffic.

**Table 5:** Communities and link prediction for “Biofuels” export graph, in 2019. Size means number of ports + number of countries in the community

Cluster #	Size	Ports	Missed potential markets
1	5 + 9	**Barcelona**	Greece, Ireland, Morocco.
		**Cartagena**	Belgium, Bulgaria, Ireland, Morocco, United Kingdom.
		**Algeciras/Cádiz**	Bulgaria, France, Greece, Ireland, United Kingdom.
		**Castellón**	Belgium, Greece, Ireland, Italy, Morocco, Netherlands.
		**Coruña/Ferrol**	Bulgaria, Greece, Italy, Morocco.
2	1 + 17	**Valencia**	
3	1 + 5	**Bilbao**	
4	1 + 1	**Alicante**	
5	1 + 1	**Huelva**	

**Fig. 3:**
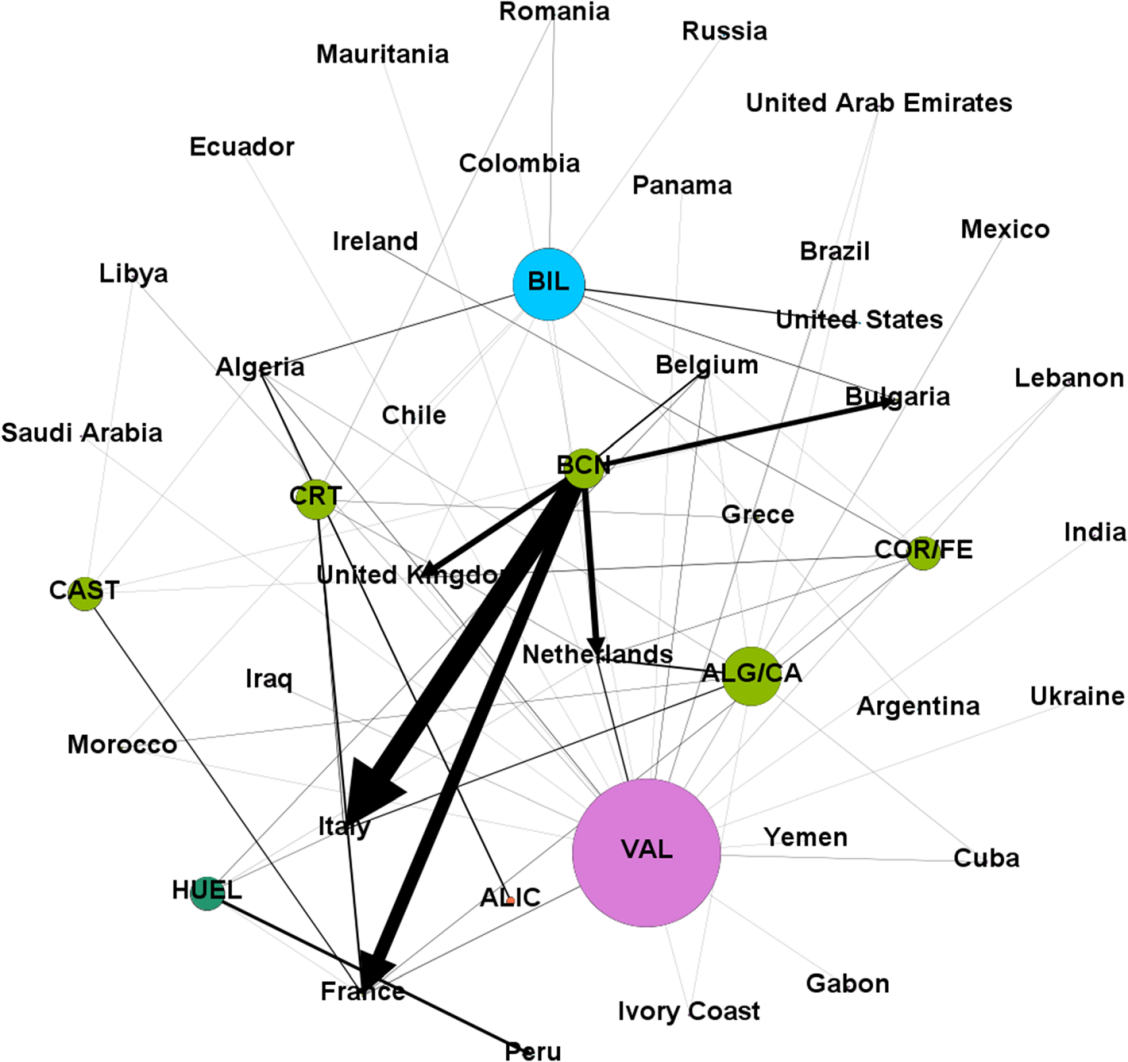
Main export links for biofuels in 2019. Nodes and arrows are proportional to the degree of the node and the weight of the link respectively. Nodes in the same community are represented with the same color

The great predominance of the port of Barcelona over the others results that unsurprisingly, the five most important links for the export of this material are those formed by the aforementioned port with Italy, France, the Netherlands, the United Kingdom and Bulgaria. It is significant that, although the high weight of the links of the port of Barcelona with the countries with which it has traffic clearly dominates the export market for this product, the port of Valencia, as mentioned above, has many links with a much smaller weight, but with many countries. This makes its degree within the graph much higher and therefore it is represented by a node of a much larger size than the other ports (Fig. 3).

### Import

#### Automobiles and their parts

The 17 vehicle manufacturing plants installed in Spain require the supply of automotive component parts for their assembly and the production of the cars that in most cases are aimed at export, as indicated above. The import of these goods is led mainly by the ports of Pontevedra, Valencia and Barcelona, adding among these three ports more than 80% of the total imports.

The ports in Pontevedra province, where the PSA Groupe is located, do not share a community with any other port as the vast majority of traffic come from France. The application of Infomap to this traffic detects a large community formed by 20 nodes, which include the ports of Valencia, Tarragona, Barcelona, Bilbao and Algeciras/Cádiz. These data and the link prediction for the ports in the cluster can be seen in Table 6.

**Table 6:** Communities and link prediction for the “Automobiles and their parts” import graph, in 2019. Size means number of ports + number of countries in the community

Cluster #	Size	Ports	Missed potential markets
1	5 + 15	**Valencia**	Italy, Poland, Taiwan, Vietnam.
		**Tarragona**	China, India, Italy, Mexico, Poland, South Africa, Sweden, Taiwan, United Kingdom, United States, Vietnam.
		**Barcelona**	South Africa, Sweden, Taiwan, United States.
		**Bilbao**	Italy, Japan, Poland, South Africa, Sweden, Thailand, United Kingdom, Vietnam.
		**Algeciras/Cádiz**	Italy, Mexico, Poland, Sweden, Taiwan, United Kingdom, Vietnam.
2	1 + 1	**Pontevedra**	
3	1 + 4	**Las Palmas**	
4	1 + 1	**Málaga**	

**Fig. 4:**
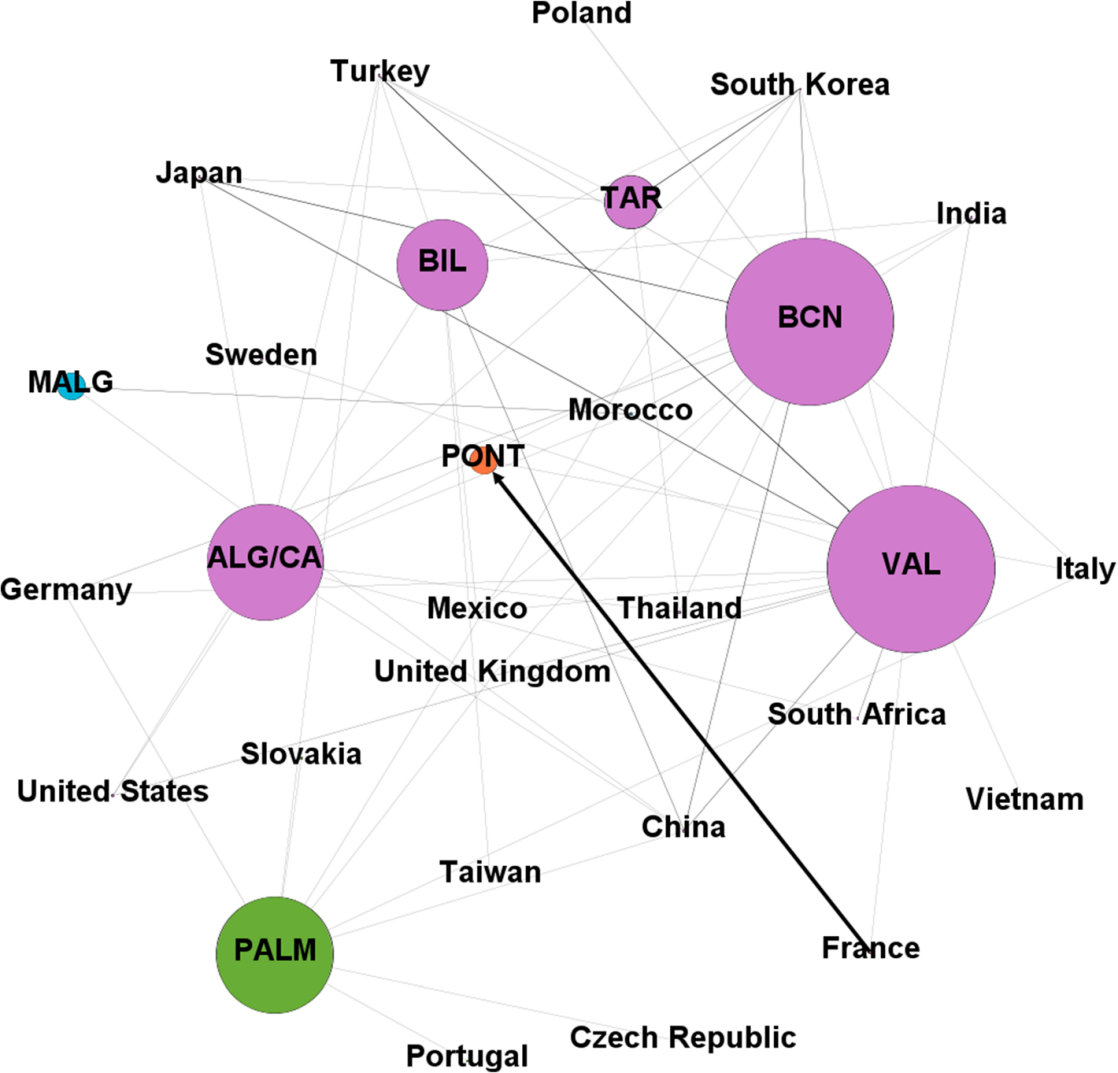
Main import links for “Automobiles and their parts” in 2019. Nodes and arrows are proportional to the degree of the node and the weight of the link respectively. Nodes in the same community are represented with the same color

Despite the predominance of the ports in Pontevedra for this traffic, and the fact of only having links with France and Italy, make the degree within the network very small (Fig. 4).

#### Biofuels

As previously mentioned, the export traffic of this product is strongly dominated by the port of Barcelona, but in the case of imports, the port of Huelva leads imports, followed by the port of Barcelona. Both ports monopolise more than 80% of total imports for this commodity. The presence of important production, storage and distribution infrastructures in the port of Huelva, helps to consolidate its leadership position.

Despite the leading position of both ports, the differences among the countries of origin of the import traffic make them appear in different communities. The port of Huelva shares a community with the ports of Algeciras/Cádiz, and the port of Barcelona shares a cluster with the ports of Coruña/Ferrol. The ports of Bilbao and Valencia appear in the third of the communities for this type of import traffic. The details of the communities and the link prediction is shown in Table 7.

**Table 7:** Communities and link prediction for “Biofuels” import graph, in 2019. Size means number of ports + number of countries in the community

Cluster #	Size	Ports	Missed potential markets
1	2 + 6	**Huelva**	
		**Algeciras/Cádiz**	China, India, Indonesia, Taiwan.
2	2 + 7	**Barcelona**	
		**Coruña/Ferrol**	Bulgaria, Denmark, France, Italy, Portugal, United Kingdom.
3	2 + 7	**Bilbao**	Barbados, Canada, South Africa, South Korea, Turkey.
		**Valencia**	Ireland.
4	1 + 2	**Tarragona**	

**Fig. 5:**
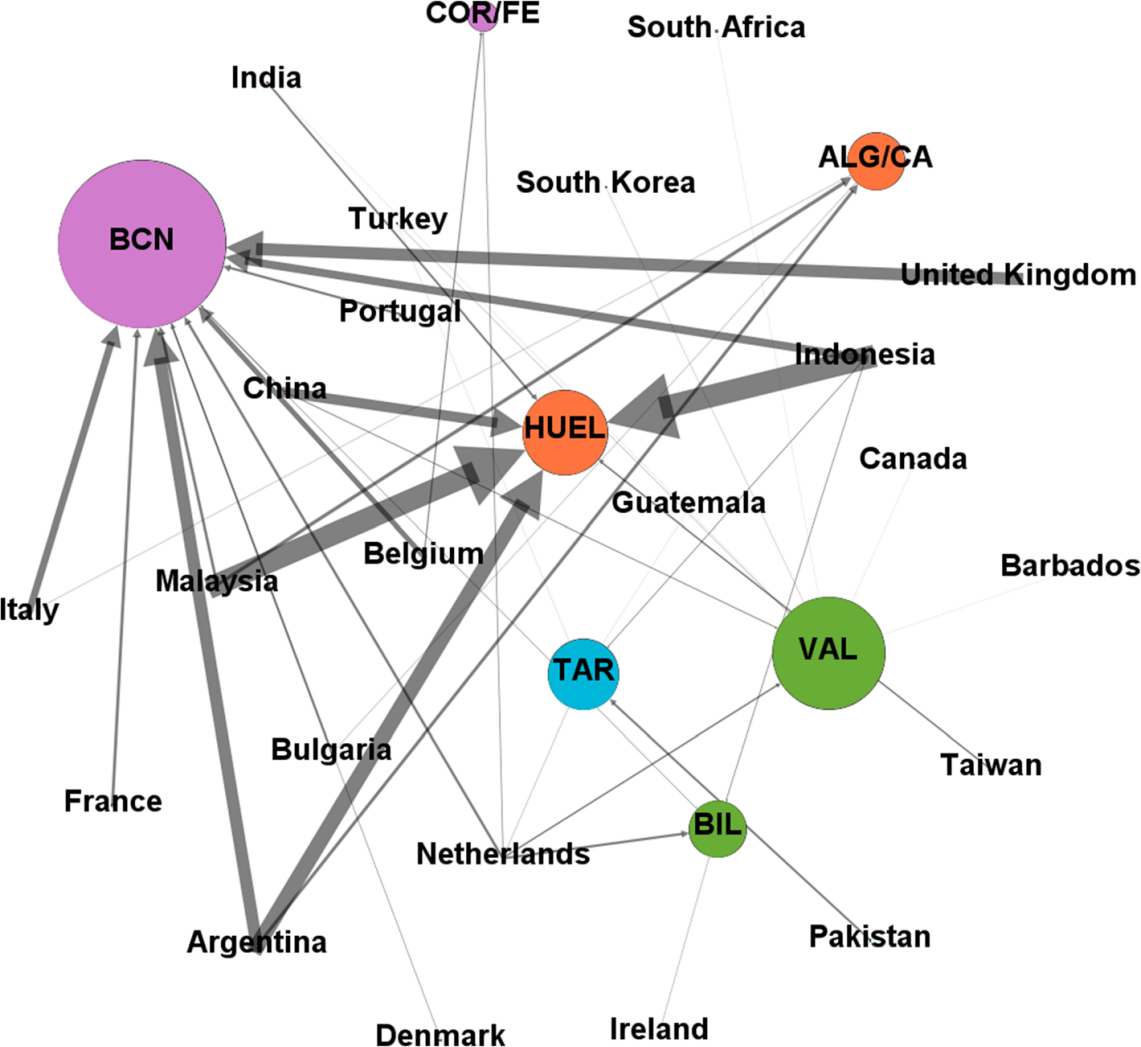
Main import links for biofuels in 2019. Nodes and arrows are proportional to the degree of the node and the weight of the link respectively. Nodes in the same community are represented with the same color

The three links with more weight have as a destination the port of Huelva, and come from Indonesia, Malaysia and Argentina. In spite of this, although with smaller weights, the port of Barcelona presents many more connections and therefore it is represented with a larger node (Fig. 5).

### Port communities identification

The procedure described in the previous section for the two types of products selected illustrate the approach proposed in this paper for the identification of port clusters. For a full analysis, it has been repeated for the import and export trade for the 38 categories of products with available data, for both the first and last years of the data set (2009 and 2019).

The application of the algorithm to all export traffic in 2009 detects 55 communities having two or more ports grouped together. The procedure for import flows detects 46 clusters having two or more ports. Regarding 2019, 58 communities with two or more ports were detected considering export flows, and 57 clusters for import flows. Based on the assumption that the more communities share two ports the more similar the traffic handled by those two ports will be, the number of times two ports share a community is calculated as a proxy of the similarity of the ports regarding their international trade.

A summary of the overall results is shown in Table 8, in which for each port the ports with which it shares more communities for the years 2009 and 2019 are listed, both for export and import, considering the 38 product categories in the data base.

**Table 8:** For each port in the study, number of times that another port belongs to the same community, taking into account the 38 product categories. Ports are grouped into brackets when they share the same number of communities. Data for export (CE) and import (CI) flows in 2009 and 2019

PORT	CE 2009	CE 2019	CI 2009	CI 2019
**ALG/CA**	3-VAL; 2-{BCN, ALM}	5-VAL; 4-BCN; 3-{ALM, CRT}	5-BCN; 4-{VAL, HUEL, SNT, CRT, BIL, SNT}	4-{VAL, BCN}; 3-HUEL
**ALIC**	1-SEV	1-{ALG/CA, ALM}	1-{ALG/CA, ALM, SEV}	2-SEV
**ALM**	2-{ALG/CA, CRT, TAR}	3-ALG/CA; 2-VAL; 1-{CRT, BCN, ALIC, SEV, MALG}	2-{ALG/CA, CRT, BCN, VAL, TAR}	1-{ALG/CA, CRT, BCN, VAL}
**BCN**	7-BIL; 4-VAL; 3-{COR/FE, PONT}	9-VAL; 5-BIL; 4-ALG/CA	8-VAL; 5-{BIL, PONT, ALG/CA, CRT, HUEL, TAR}	18-VAL; 10-{BIL, COR/FE}
**BIL**	7-BCN; 5-COR/FE; 2-{VAL, PAS}	6-VAL; 5-BCN; 4-COR/FE	5-{BCN, VAL, PONT}	11-VAL; 10-BCN; 7-PONT
**CRT**	3-TAR; 2-{COR/FE, HUEL, VAL, ALM}	4-VAL; 3-{COR/FE, BCN, ALG/CA, BIL}	5-BCN; 4-ALG/CA; 3-{TAR, HUEL, VAL}	7-BCN; 6-{VAL, TAR}; 5-CAST
**CAST**	1-{BCN, TNR, TAR, MOT}	3-{BCN, VAL}; 2-{COR/FE, CRT}	3-{TAR, BCN}; 2-VAL	5-{CRT, TAR}; 3-{BCN, VAL, COR/FE, BIL}
**CEU**	**–**	**–**	**–**	1-GIJ/AV
**COR/FE**	5-BIL; 3-{BCN, TAR, HUEL}	4-BIL; 3-CRT; 2-{BCN, ALG/CA, CAST}	3-{VAL, ALG/CA} 2-{BIL, BCN, PONT, TAR, SNT}	10-BCN; 7-VAL; 6-PONT
**GIJ/AV**	1-{HUEL, ALG/CA}	3-{SNT, VAL, BIL}	1-ALG/CA	6-VAL; 4-{BIL, BCN, HUEL}
**HUEL**	3-COR/FE; 2-CRT; 1-GIJ/AV	3-TAR; 2-ALG/CA;1-{CRT, GIJ/AV, BIL, BCN}	5-BCN; 4-ALG/CA; 3-{CRT, TAR}	6-TAR: 4-{CRT, GIJ/AV}
**PALM**	1-{BIL, COR/FE, SEV, TNR}	1-PONT	5-TNR; 1-SEV	6-TNR; 2-{PONT, COR/FE}
**LISB**	**–**	1-{ALG/CA, BCN}	–	2-{VAL, BCN}1-{ALG/CA, PONT}
**MALG**	2- ALG/CA; 1-SEV	3-BCN;2-{SEV, CRT, VAL}	1-{BCN, CRT, VAL, ALM, ALG/CA, MOT}	2-SEV; 1-{CRT, BCN, VAL, PONT}
**MALL**	**–**	–	2-TAR; 1-{VAL, TNR}	2-VAL; 1-{TNR, SNT, PONT}
**MEL**	**–**	–	1-{VAL, TNR, PAS}	–
**MOT**	1-CAST	**–**	2-VAL; 1-{CAST, SEV, TAR, PAS, STN, MALG}	2-{VAL, STN, PAS, PONT}
**PAS**	2-{BCN, BIL, COR/FE}	2-BCN; 1-{PONT, CRT, ALG/CA}	2-BCN; 1-{ALG/CA, CRT, SEV, SNT, VAL, MOT, MEL}	5-{BCN, BIL}; 4-{COR/FER, VAL}
**PONT**	3-BCN;2-{COR/FE, VAL}	1-{BCN, PAS, MALG, CRT, PALM, GIJ/AV, CAST, STN}	5-{BIL, BCN}; 4-VAL	9-VAL; 8-BCN; 7-BIL
**SNT**	1-{BIL, BCN, VAL, TAR, SEV, PAS}	3-{BIL, GIJ/AV}; 1-{BCN, VAL, PONT}	4-{BCN, VAL, TAR, ALG/CA}	4-{BCN, VAL, PONT, TAR, COR/FE}
**SEV**	1-{BCN, VAL, ALG/CA, TAR, SNT, COR/FE}	2-{VAL, MAL}; 1-{BCN, CRT, ALG/CA, TNR, ALM, CAST}	2-BCN; 1-{VAL, CRT, TNR, ALM, CAST, PAS, BIL, TAR, PALM, ALIC, MOT}	3-{BCN, COR/FE, BIL, TAR}
**TAR**	3-{COR/FE, CRT}; 2-{BCN, ALM}	3-HUEL; 2-BCN; 1-{COR/FE, CRT}	5-{BCN, VAL}; 4-SNT	8-{BCN, VAL}; 6-{HUEL, CRT, BIL}
**TNR**	1-{TAR, BCN, CAST, PALM}	1-SEV	5-PALM; 2-{BCN, TAR}	6-PALM; 3-{BCN, VAL}
**VAL**	4-BCN; 3-ALG/CA; 2-{COR/FE, CRT, BIL, PONT}	9-BCN; 6-BIL; 5-ALG/CA	8-BCN; 5-{TAR, BIL}; 4-{ALG/CA, PONT, SNT}	18-BCN; 11-BIL; 9-PONT

Although the Portuguese ports have been included in this study and it may be interesting to consider the routing through them of certain goods in the south-west of Spain, it can be noted that only the port of Lisbon shares communities with Spanish ports in the year 2019, while in 2009 it did not share any community with another Spanish port.

## Discussion

López-Bermúdez et al. [44] proposed a classification of the 28 Spanish port authorities in a time period (2011 to 2018) similar to that studied in our research. Although this paper does not differentiate between import and export flows nor analyse the different categories of products beyond their mode of presentation, it is interesting to note the coincidence of some of the ports listed in the above classifications and the communities that share the ports in this study. Ports of Algeciras, Barcelona and Valencia (along with others) are included within the ports specialised in containerised cargo and share a large number of communities.

The fine grain analysis of our study and the different results for import and export flows means that a greater number of communities do not always coincide with the general classification of ports proposed in the above-mentioned paper, but similar results can be seen in some cases, such as in the case of solid bulks, which includes the ports of Coruña, Huelva, Motril, Cartagena and Tarragona. The calculation of the communities to which ports and countries belong for a certain type of cargo provides a reliable indicator of the possibilities of competition or collaboration for this particular traffic.

According to the official statistics of Puertos del Estado [51], in 2009 the top five positions of total traffic in the Spanish port system were occupied by the ports of Algeciras, Valencia, Barcelona, Bilbao and Tarragona. In 2019 the total traffic of goods in the Spanish port system was occupied in its first four places by the same ports of 2009 with the port of Cartagena (which ranked eighth in 2009) now in fifth place, replacing the port of Tarragona.

As mentioned in the previous section, once we know the communities to which the ports of the Spanish port system belong to for each of the goods, it can be inferred that the more communities two ports share, the more similar these ports will be, and therefore the more possibilities they will have to establish cooperation mechanisms among them.

Reviewing the results in Table 8, it can be seen that the highest number of communities shared by any two ports occur in the four largest ports of the Spanish port system. For instance, in 2019 Barcelona shared 18 importing communities with Valencia and 10 with Bilbao, and Valencia 11 with Bilbao. Algeciras shared with Barcelona and Valencia most of their communities. Also, the geographical location seems to be relevant. Note also the great similarity in the importations of the Canarian ports (Tenerife and Las Palmas) which share a large number of communities for import traffic in the 2 years of the time series considered.

To graphically represent the membership of the ports of the Spanish port system to the communities detected by the Infomap algorithm for each of the traffics, some non-directed networks have been created that have the ports themselves as nodes. There is a link between a pair of nodes if they share at least one community among them for the year of study and the type of the traffic being analysed, and this link will have as weight the number of communities these ports share. The size of the nodes will be plotted in accordance with the degree of each node within the graph (that is, the number of competitors), while the width of the links will be represented as a function of their weight (that is, the intensity of competition between both ports measured as the number of products in which they compete).

**Fig. 6:**
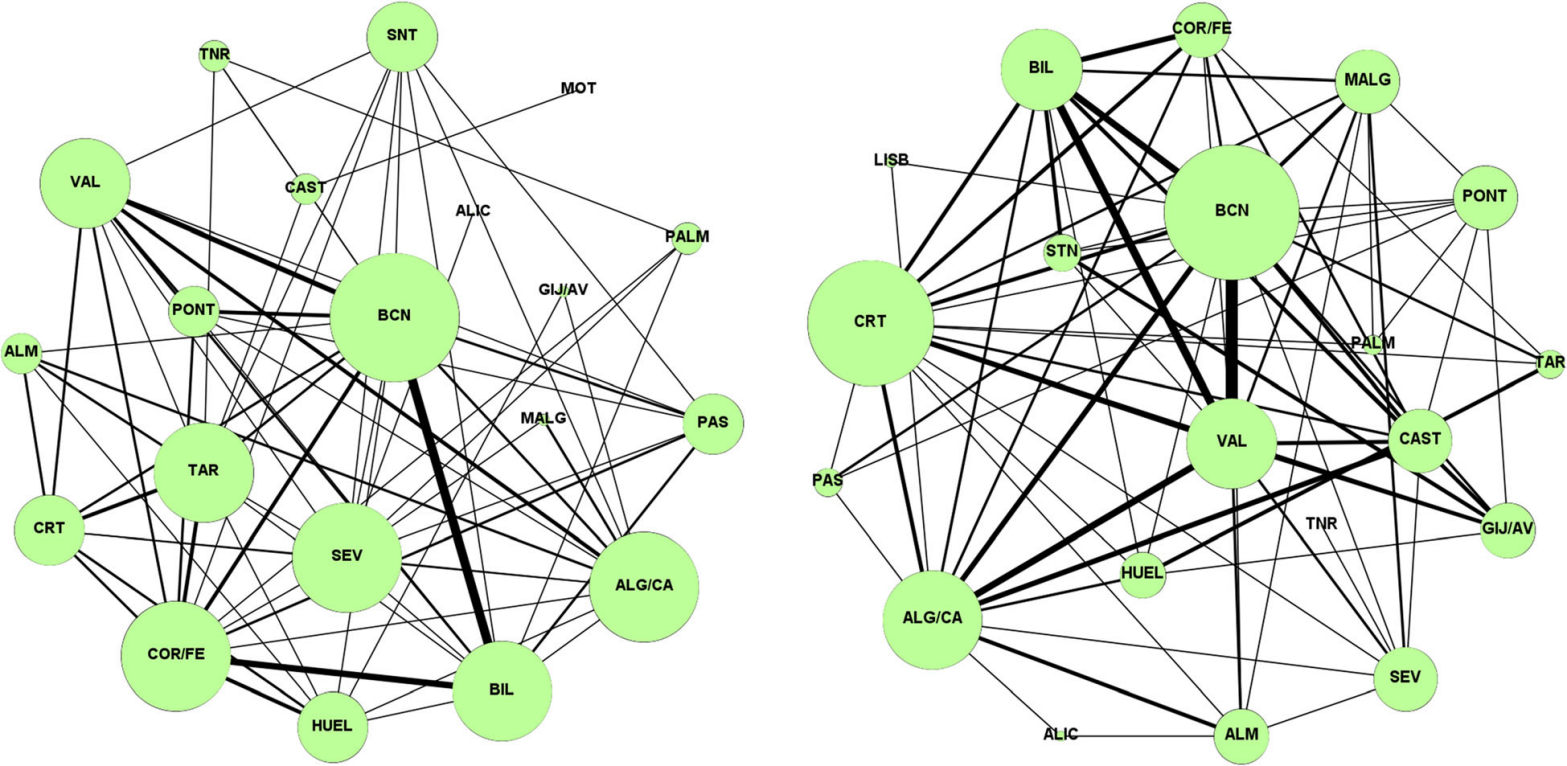
Graphs representing the membership of pairs of ports to the same community for export flows in 2009 (left) and 2019 (right). The size of the nodes is proportional to their degree, and the thickness of the links is proportional to the number of communities shared by both ports

Figure 6 shows the ports that share export traffic communities for 2009 and 2019. It can be observed for 2009 the high degree of the port of Barcelona, meaning that it shares communities (similar trading) with a large number of ports, notably with Valencia and Bilbao. A similar degree is noted for the ports of Algeciras/Cádiz, in this case with much smaller weight of the links (many “competitors” but for less products).

Comparing with 2019, Barcelona is still the largest node but it is notable the increment of the width of the links among Barcelona, Valencia and Bilbao. The competition among this “big 3” is much more intense 10 years later. There is a greater number of ports sharing a community than in 2009 and the increase in the grade of the port of Cartagena is noteworthy. It is easy to visually distinguish the figure as the weight and number of links has risen considerably in 2019 in comparison with the initial situation in 2009.

**Fig. 7:**
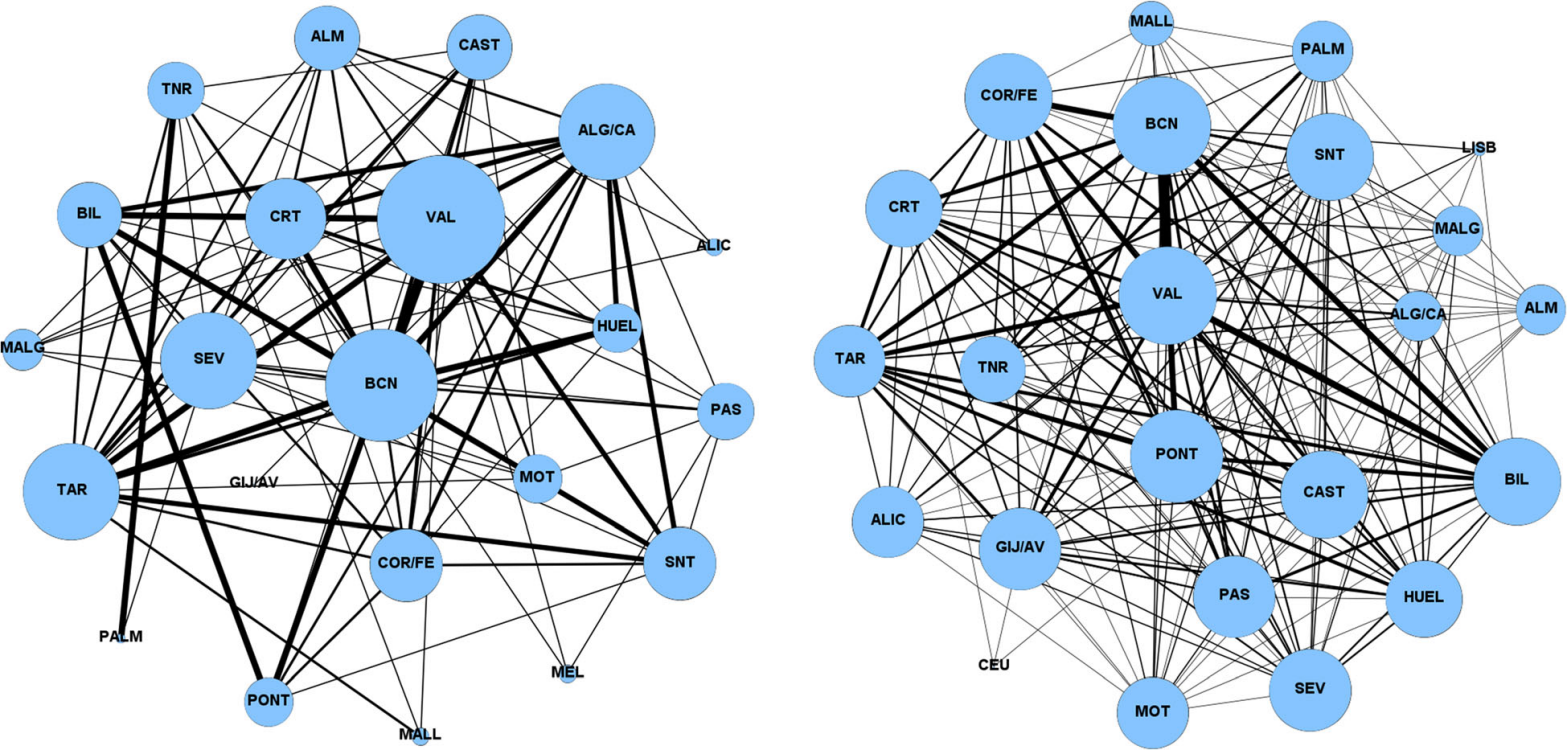
Graphs representing the membership of pairs of ports to the same community for import flows in 2009 (left) and 2019 (right). The size of the nodes is proportional to their degree, and the thickness of the links is proportional to the number of communities shared by both ports

The evolution of the import was smoother than in the case for exports. As shown in Table 2, the total number of links in the graphs show growth of only a 14% against the 44% of the exports’ links. That is, there was a more intense export action than import in the ports’ strategy. Figure 7 shows the evolution of the communities that ports share in 2009 and 2019. For 2009, several ports with a similar degree visually stand out in the figure, namely the ports of Valencia, Barcelona, Algeciras/Cádiz. 2019 shows a large number of links and a degree of the nodes with not as many variations as in the previous cases. It can be noteda large number of links with a high weight, and a trend towards homogenisation in the degree of nodes in relation to 2009.

The most outstanding aspects of the time evolution of export flows include the increase in the degree (greater number of times sharing communities with other ports) of Cartagena, Malaga and Gijon/Avilés, and the decrease in the degree of the ports of Tarragona and Coruña/Ferrol. As for the import flows, it is worth noting a greater number of links than the export flows (there is a larger number of communities formed by two or more ports) and that the degree of the nodes is more similar in this case than in the export flows, in which there are great variations in the degree of the nodes, both for 2009 and 2019.

## Conclusions

From the best knowledge of the authors, this work is a pioneer in analysing the international traffic to categorise the port system of a country from the perspective of complex networks, with a fine grain comparison that may allow the analysis of business opportunities for the ports included in the survey.

The communities that a port shares with the ports in its area, and the countries that are part of the cluster with them, can provide valuable information for the subsequent analysis of the causes that have produced this evolution and thus decide the most appropriate strategies for the management of their ports.

The observation of the figures representing the communities for the goods surveyed in the previous sections in the Spanish case show that for certain commodities some ports dominate the import or export market with traffics from (or to) a very small number of countries. The fact of having few large links (supply of a certain product with origin or destination in a small number of countries) can lead to a problem of vulnerability for the port, given that an event that disrupts connectivity between a country with strong connections to a given port, could cause a critical problem in the supply chain and the shortage of the traffic involved. For this reason, it has been preferred to include both the grade of the ports and the weight of the links in the graphic representation of the selected traffics.

One of the most interesting tools for traffic analysis is the “link prediction” for each type of traffic, as illustrated in the selected traffic of section 4. The absence of traffic with a certain country for ports that share a community allows us to identify countries that can be a commercial objective (potential markets) for the capture of the studied cargo.

The sum of the membership of the ports to the communities described above and its graphical representation, allows to verify for both export and import flows, the evolution of the ports during the period of time covered by the study. In this graphic representation can be noted the similarities between the ports with more movement of goods within the Spanish port system: Valencia, Barcelona and Algeciras, located the first two on the Mediterranean façade and in the Strait of Gibraltar the third. Far from there in the north facade, the port with more movement of tonnes and more regular lines, Bilbao, also shares a great number of communities with these ports.

These ports are included as “Core Ports” in the two Core Network Corridors of the TEN-T Core Network crossing the Iberian Peninsula, namely the Mediterranean and the Atlantic Corridors. Belonging to this network means benefiting from public aid and being considered strategic nodes within the European supply chain. This network also includes the Portuguese ports included in this study, and the ports of Cartagena and Tarragona.

The evolution of the TEN-T network is still in the design process and there are initiatives to include part of the ports on the Cantabrian coast in the Core network. There is no doubt that this decision will affect the future of the ports and the regions of their areas of influence due to the fact that the largest public investments will be concentrated in the Core ports of the European network.

It must be noted that the ports that appear together in one of the detected communities are not necessarily competitors. There is no doubt that large ports, having a greater number of lines than small ports will tend to appear in a greater number of communities than small ports among themselves. Ports with very different sizes and business models may appear in the same community if they have similar traffic (countries of origin or destination) for a given commodity type. However, similar traffic must identified and placed in context with the rest of the information available on that cargo before taking further steps on possible collaborations or exploring business opportunities for a specific cargo in a particular port.

The creation of communities for specific traffics described in this study should be understood as a high-level functional consultancy tool for port managers which, along with the rest of the available tools, can assist decision-making in such a competitive and evolving environment as the maritime sector.

This study aims to be a valuable tool that can be incorporated into the rest of the information available for port managers and global decision-makers in order to take the most appropriate decisions in each case.

As future research, it could be interesting to extend this research to other regions or to other ports with different temporal scopes. This would require the availability of systematised and standardised initial data in the same way as the dataset used in this research.

An interesting issue to study could be to test whether the regulation, governances and even the port culture itself have influence in the communities formed. Once used our approach (in the Spanish system or any other area) to identify the clusters, a post analysis could check if those characteristics of each port are influencing the communities formation. In any case, the lack of data has proven to be one of the most important impediments to the study of maritime traffic in general.

## Data Availability

The authors provide under request the filtered data used in this study.
